# Traditional Chinese Medicine JianPiHuaTan formula improving quality of life and survival in patients with colorectal cancer through RAS/RAF downstream signaling pathways

**DOI:** 10.3389/fphar.2024.1391399

**Published:** 2024-06-20

**Authors:** Jian He, Guojun Li, Yu Wu, Tong Zhang, Mingjiang Yao, Mingxuan Zang, Jianhua Zou, Jinjie Song, Liusheng Li, Qian Chen, Guang Cao, Linlin Cai

**Affiliations:** ^1^ GCP Center, Guang’anmen Hospital, China Academy of Chinese Medical Sciences, Beijing, China; ^2^ Department of Oncology, Xiyuan Hospital, China Academy of Chinese Medical Sciences, Beijing, China; ^3^ Institute of Basic Medical Sciences of Xiyuan Hospital, China Academy of Chinese Medical Sciences, Beijing Key Laboratory of Pharmacology of Chinese Materia, Beijing, China; ^4^ Department of Oncology, Beijing Hospital of Integrated Traditional Chinese and Western Medicine, Beijing, China; ^5^ Thorgene Co., Ltd., Beijing, China; ^6^ Department of General Surgery, Beijing Anzhen Hospital, Capital Medical University, Beijing, China

**Keywords:** traditional Chinese medicine, colorectal cancer, PDX models, transcriptome, therapeutic effects

## Abstract

**Objective:**

JianPiHuaTan Formula (JPHTF), a traditional Chinese medicine (TCM), has been utilized as an adjunctive therapy for colorectal cancer (CRC). The study aims to evaluate the potential clinical benefits of JPHTF and its effectiveness in inhibiting tumor growth.

**Methods:**

300 stage II/III CRC patients and 412 advanced CRC patients were enrolled to verify the clinical value of JPHTF in CRC treatment. Furthermore, CRC patient-derived xenograft (PDX) mice were utilized to investigate the regulatory mechanisms of JPHTF.

**Results:**

JPHTF significantly improved abdominal distension, shortness of breath, drowsiness, loss of appetite, sleep, and tiredness in stage II/III CRC patients, thereby improving their quality of life. Simultaneously, JPHTF served as a supportive therapy in extending the overall survival (OS) of stage IV CRC patients with RAS/RAF mutations undergoing chemotherapy. Additionally, JPHTF effectively impeded tumor progression in CRC PDX models with RAS mutation, accompanied by a reduction in tumor cell content in the JPHTF group. Transcriptomic analysis revealed the involvement of the Hippo and Hedgehog signaling pathways in JPHTF-mediated CRC inhibition. Furthermore, mice in the JPHTF group exhibited increased immune cell infiltration.

**Conclusion:**

These findings suggested that JPHTF may inhibits tumor growth in CRC with RAS mutation by modulating RAS/RAF downstream signaling pathways, specifically the Hippo and Hedgehog signaling, leading to increased immune cell infiltration.

## 1 Introduction

CRC is a common malignant tumor of the digestive system. Both cellular and animal functional analyses, as well as clinical studies, indicate the anticancer effects of TCM in the treatment of CRC. TCM can disrupt the survival environment of cancer cells, promote apoptosis, inhibit tumor cell proliferation and metastasis, and modulate the immune system to achieve antitumor efficacy within the tumor microenvironment (Xu et al., 2015; Yan et al., 2017; Zhang et al., 2020a; Sun B. et al., 2021; Sun Q. et al., 2021). The regulation and mechanisms of the intestinal microbiota in colorectal cancer are beneficial for disease treatment, with current research suggesting that TCM formulations, individual herbal medicines, and TCM monomeric compounds can be used to treat CRC through the modulation of the intestinal microbiota (Zhao et al., 2021). Additionally, TCM can be used in conjunction with other chemotherapy drugs to reduce adverse reactions induced by chemotherapy, significantly improving the quality of life for cancer patients (Chen et al., 2021; Wang et al., 2021). In comparison to the precision treatment models of modern medicine, TCM adopts a precision treatment approach characterized by overall regulation of the body’s systems and individualized syndrome differentiation, demonstrating its advantages throughout various stages of CRC progression.

The combination of TCM and adjuvant chemotherapy can reduce the occurrence of toxic side effects, such as hematological toxicity, gastrointestinal reactions, bone marrow suppression, and peripheral neuropathy, while also regulating immune function and enhancing patient quality of life (Kummar et al., 2011; Li et al., 2021). Treatment with TCM in conjunction with or following chemotherapy can prolong disease-free survival (DFS) for CRC patients, particularly reducing the risk of recurrence and metastasis in stage II/III patients (Yang et al., 2008; Xu et al., 2017; Wang et al., 2020). Furthermore, the differentiation and treatment based on syndrome differentiation in traditional Chinese medicine play a key role in clinical research. Spleen deficiency syndrome predominates in the syndrome differentiation of colorectal cancer and has become one of the universally recognized basic syndromes in the diagnosis and treatment of colorectal cancer within TCM (Sun L. et al., 2021). The combination of TCM syndrome differentiation and specific disease identification is also an important indicator for assessing the effectiveness of tumor treatment in traditional Chinese medicine.

Transcriptome studies provide a deeper understanding of gene expression and regulatory mechanisms at the RNA level, holding crucial significance for the guidance of disease occurrence, development, and treatment. TCM treatment, derived from the medical system of Chinese civilization through extensive clinical practice and accumulated experience, uniquely focuses on comprehensive regulation of the body’s internal environment to improve disease symptoms and enhance patient quality of life (Zhang et al., 2020b). Changes in the internal environment may affect the expression and regulation of specific genes, thereby influencing multiple aspects of cellular signal transduction and metabolic pathways (Chen et al., 2020; Zhang et al., 2021). Therefore, conducting research on the impact of TCM treatment on the transcriptome helps us gain insight into the efficacy and mechanisms of TCM treatment, providing a scientific basis for the clinical application of TCM.

Our study aims to investigate the efficacy of JPHTF treatment, by evaluating the therapeutic effects of JPHTF in cohorts of stage II/III and late-stage CRC patients, and further validating the anticancer effects of JPHTF in a CRC PDX models with RAS mutation.

## 2 Materials and methods

### 2.1 Patients data

This retrospective study aimed to evaluate the relative efficacy of JPHTF treatment in CRC patients. The cohorts included stage II/III and IV CRC patients. Written informed consent was obtained from each participant. For the stage II/III CRC cohort, 350 patients were recruited from 8 centers. Eligible patients were aged 18 years or older, including high-risk stage II and III CRC patients who had undergone surgery and completed adjuvant chemotherapy within 3 months without tumor recurrence or metastasis. Patients with a history of previous or concurrent malignancies, excluding cured cases of skin basal cell carcinoma and cervical carcinoma *in situ*, as well as those with known severe cardiovascular, hepatic, or renal diseases, were excluded. Additionally, individuals with any conditions that could jeopardize patient safety and compliance with the study, such as pregnancy, depression, bipolar disorder, obsessive-compulsive disorder, or schizophrenia, were also excluded. 10 participants were excluded from the study due to missing follow-up data on DFS. Furthermore, TCM syndrome, ESAS and ECGO data were not completely acquired for 40 subjects during the following visit. A total of 300 cases were included in this analysis, with 147 patients received placebo treatment (1/10 of the trial dose) and 153 patients received JPHTF treatment (Supplementary Figure S1). These data were collected from December 2017 to May 2021, with the last follow-up conducted in January 2022. The study was approved by the Ethics Committee of Xiyuan Hospital, China Academy of Chinese Medical Sciences (2018XLA043-2). Among them, 147 patients received placebo treatment (1/10 of the trial dose) and 153 patients received JPHTF treatment. The primary endpoint was to assess the improvement in quality of life, while secondary endpoints included recurrence rate and DFS.

For the stage IV CRC cohort, 414 patients from 9 centers diagnosed with stage IV CRC were enrolled. Eligible participants were aged 18 years or older, had previously received treatment with oxaliplatin, fluorouracil, and irinotecan, either sequentially or concurrently, and experienced disease progression or were intolerant to further chemotherapy, with a life expectancy of more than 3 months. Patients with a history of previous or concurrent malignancies, excluding cured cases of skin basal cell carcinoma and cervical carcinoma *in situ*, as well as those with severe cardiac, hepatic, or renal complications, were excluded. Additionally, patients with intestinal obstruction, requiring parenteral nutrition due to malabsorption, or with active peptic ulcer disease were also excluded. Two participants were excluded due to missing follow-up data on survival outcomes. A total of 412 patients were included in the study, with data collection spanning from February 2016 to May 2019, with the last follow-up conducted in November 2019. The study was approved by the Ethics Committee of Xiyuan Hospital, China Academy of Chinese Medical Sciences (2016XLA121-3). The patients were divided into three groups: Western medicine (WM) (*N* = 120), TCM (*N* = 138), and Integrated Traditional Chinese and Western medicine (ITWM) (*N* = 154) (Supplementary Figure S2). The TCM treatment group received treatment with JPHTF. The WM and ITWM groups underwent conventional subsequent line therapy, adhering to the guidelines established by the NCCN or CSCO, in addition to exploratory treatments that are not necessarily guideline-dependent, encompassing chemotherapy, targeted therapy, and radiotherapy for metastatic lesions. Retrospective survival analysis was used to objectively assess the adjuvant therapeutic effect of JPHTF on patients with stage IV CRC.

### 2.2 Preparation of Chinese Medicine formula

The JPHTF, developed in secret by the Oncology Department of the China Academy of Chinese Medical Sciences Xiyuan Hospital. This formula consists of 30g of ASTRAGALI RADIX, 10g of GINSENG RADIX ET RHIZOMA, and six other botanical drugs: 6g of ARISAEMATIS RHIZOMA, 10g of LIGUSTRI LUCIDI FRUCTUS, 30g of PORIA, 10g of EPIMEDIUM FOLIUM, 10g of CURCUMAE LONGAE RHIZOMA, and 15g of HERBA SALVIA CHINENSIS. The names of these botanical drugs in Chinese script, English translation, and origin are listed in Table 1. Supplementary Table S1 lists the drug—extract ratio and other basic pharmaceutical parameters. The criteria for the quality of the botanical drugs in JPHTF were in accordance with the 2020 Chinese Pharmacopoeia. The concentrated granules of JPHTF were prepared and produced by Beijing Tcmages Pharmaceutical Co., Ltd. The extraction process of JPHTF granules includes the following steps: using water to decoct and boil the botanical drugs, and the volatile oil of CURCUMAE LONGAE RHIZOMA also needs to be collected after decoction and boiling. The extracted liquid undergoes centrifugal filtration, low-temperature vacuum concentration to form a paste, and is then processed into granules using spray drying and dry granulation techniques (Supplementary Table S2). Each botanical drug in the formula is produced in accordance with the standards of the Chinese Pharmacopoeia or the National Drug Standard for Traditional Chinese Medicine Formula Granules, as well as the internal quality control standards of Beijing Tcmages Pharmaceutical Co., Ltd, and has passed quality control testing, including testing for heavy metals and harmful elements, microbial contamination limits, and residual pesticides. Additionally, production of the formula has met the National Drug Standard of the National Medical Products Administration (NMPA), with ARISAEMATIS RHIZOMA also meeting the Beijing Traditional Chinese Medicine Formula Granule Production Standards.

**TABLE 1:** Contents of JPHTF.

English translation	Chinese simplified script	English name	Latin name	Family	Drug name	Medicinal part	Place of origin of the botanical drug
Huang-Qi	蒙古黄芪	Mongolia Astragalus	*Astragalus mongholicus Bge.(Hsiao)*	Fabaceae	ASTRAGALI RADIX	Root	Longxi County, Dingxi, Gansu Province, China
Ren-Shen	人参	Ginseng	*Panax ginseng C.A.Mey.*	*Araliaceae*	GINSENG RADIX ET RHIZOMA	Root and Rhizome	Fusong, Baishan City, Jilin Province, China
Tian-Nan-Xing	天南星	Arisaema heterophyllum Blume	*Arisaema erubescens(Wall.)Schott.*	Araceae	ARISAEMATIS RHIZOMA	Tuber	Luquan Yi and Miao Autonomous County, Kunming, Yunnan Province, China
Nv-Zhen	女贞子	Ligustri Lucidi Fructus	*Ligustrum lucidum Ait.*	Oleaceae	LIGUSTRI LUCIDI FRUCTUS	Fruit	Yiyang County, Luoyang City, Henan Province, China
Fu-Ling	茯苓	Poria cocos	*Poria cocos(Schw.)Wolf*	Polyporaceae	PORIA	Sclerotium	Yuexi County, Anqing City, Anhui Province, China
Yin-Yang-Huo	淫羊藿	Epimedium	*Epimedium brevicornu Maxim.*	Berberidaceae	EPIMEDIUM FOLIUM	Leaf	Li County, Longnan, Gansu Province, China
Jiang-Huang	姜黄	Turmeric	*Curcuma longa L.*	Zingiberaceae	CURCUMAE LONGAE RHIZOMA	Rhizome	Qianwei County Leshan City, Sichuan Province, China
Shi-Jian-Chuan	石见穿	Salvia chinensis	*Salvia chinensis Benth*.	Lamiaceae	HERBA SALVIA CHINENSIS	Herba	Jinzhai County, Lu’an City, Anhui Province, China

The compounds of JPHTF was characterized by A HPLC apparatus (Agilent 1200) coupled with a G1315B DAD detector, a G1311A solvent dispensation module, a G1329A autosampler, a G1316A column furnace, a G1322A degasser. A MC-Pack ODS-AQ (250 × 4.6 mmL.D.S-5 μm) C18 column was employed to partition the sample at a flow velocity of 1.0 mL/min. Throughout the procedure, thermal equilibrium was persevered at 40°C. The mobile phase consisted of 0.1% formic acid (A) and acetonitrile (B). A gradient elution procedure, from which at least three different gradient methods were optimized (see Supplementary Figures S1–S4 and Supplementary Tables S1–S4), was set at: from 0 to 15 min, 0% B; from 15 to 65 min, 0%–40% B. A representative chromatogram was obtained at a wavelength 260 nm. Based on the optimizing method, compounds with polarities from water-solubility to lipid solubility in JPHTF were entirely eluted.

### 2.3 CRC PDX models

Twenty female NTG mice (6–8 weeks old, Spfbiotech, Beijing) were acclimated for 1 week (imodels Biotechnology). To establish PDX models, we digested fresh tumor tissues from colorectal cancer with known *NRAS* mutation into single-cell form, named human colorectal cancer cell CR1398. Subsequently, these single cells were injected subcutaneously into the right shoulder of each mice, with a cell inoculum of 3 × 10^5^ cells in a volume of 0.1 mL. Tumor volume and body weight were measured weekly until the average tumor volume reached approximately 80–100 mm^3^. Based on tumor volume and body weight, the mice were randomly divided into two groups, with 10 mice per group. Treatment was initiated immediately after grouping. The JPHTF treatment group received a dosage of 3.5 g/kg, dissolved in ddH_2_O to a concentration of 350 mg/mL. Administration was carried out orally via gavage (p.o.). The start date of administration was denoted as day 0. No treatment was administered to the blank group. Throughout the treatment period, body weight, tumor length, and width were measured biweekly. Once a noticeable difference in tumor volume between the two groups emerged after 1 week, drug administration was ceased, and the tumors were excised from the mice. Tumor volume was calculated using the formula: 0.5*Length*Width^2^ (Zhang et al., 2019). The study was approved by the Institutional Animal Care and Use Committee (IACUC) of imodels (Beijing) Biotechnology Co., Ltd. [IACUC-003-2023(1)-0104].

### 2.4 Histological examination and PHH3 immunohistochemical staining

Tissues freshly harvested from 6 mice, including 3 from the untreated control group and 3 from the JPHTF treatment group, were fixed in 10% formalin and embedded in paraffin for subsequent histological analysis. Hematoxylin and eosin (H&E) staining was performed following standard protocols (Cardiff et al., 2014). Immunohistochemistry was carried out on 2 or 3-μm-thick consecutive sections using an anti-PHH3 rabbit monoclonal antibody (Dilution: 1:100, BioLynx # BX50087). The staining procedure involved pretreatment with Tris-EDTA buffer (pH = 9.0), incubation of the primary antibody at room temperature (18°C–25°C) for 30 min, followed by incubation with the diluted antibody at 37°C for 32 min. Quality assurance of immunolabeling was based on the unambiguous staining of mitotic figures (MFs) in the tumor. PHH3-positive counts were performed on the stained slides using a multi-head microscope.

### 2.5 RNA sequencing and gene expression analysis

The tumors from both the control group and the JPHTF treatment group were frozen in liquid nitrogen immediately after removal for RNA isolation. Three independent replicates for each group were analyzed. RNA extraction was performed using the RNeasy Mini Kit (Qiagen Inc., Germantown, United States). Subsequently, RNA libraries were constructed using the TruSeq RNA Exome kit (Illumina, Inc., San Diego, CA, United States). RNA-sequencing was conducted using the NovaSeq 6000 platform (Illumina Inc., San Diego, CA, United States). The obtained sequencing data were subjected to quality control filtering using the NGSQCToolkit (v2.3.3) to eliminate reads with low quality bases (>15% of bases with a quality score ≤19) and reads containing sequencing adapters. The clean reads were then aligned to the reference genome hg19 using Bowtie software (version 2.2.4) with default parameters. Gene expression levels were quantified as reads per kilobase per million reads (FPKM) using Cufflinks software (version 2.2.1) with default settings. Differential expression analysis was performed using the R package limma, using a log2 (FoldChange) ≥1 and *p*-value ≤0.05 as the threshold. To assess the enrichment of biological pathways, Kyoto Encyclopedia of Genes and Genomes (KEGG) pathway analysis was conducted using the cluster Profiler package in R (Yu et al., 2012; Kanehisa et al., 2017). Gene Set Enrichment Analysis (GSEA) (Subramanian et al., 2005) was performed using the “gsva” R package. Enrichment results with |NES| > 1, nominal *p*-value <0.05, and false discovery rate (FDR) q-value <0.25 were considered statistically significant. Immune infiltration was estimated using the ESTIMATE and CIBERSORT algorithms.

### 2.6 Statistical analysis

The statistical analysis was conducted using SPSS Statistics 25 (IBM, New York City, NY, United States) and R-4.2.2. Unpaired *t*-tests or Wilcoxon rank-sum tests (for 2 groups) and Kruskal-Wallis tests (for >2 groups) were used to compare PHH3-positive counts and immune infiltration in the untreated control group (G1) and the JPHTF treatment group (G2) groups, as well as EASA scores at different follow-up visits. Chi-square or Fisher’s exact tests were employed to compare TCM symptom classification ratings scale. A *p*-value of 0.05 or less was considered statistically significant.

## 3 Results

### 3.1 Chemical profile of JPHTF formula

**FIGURE 1:**
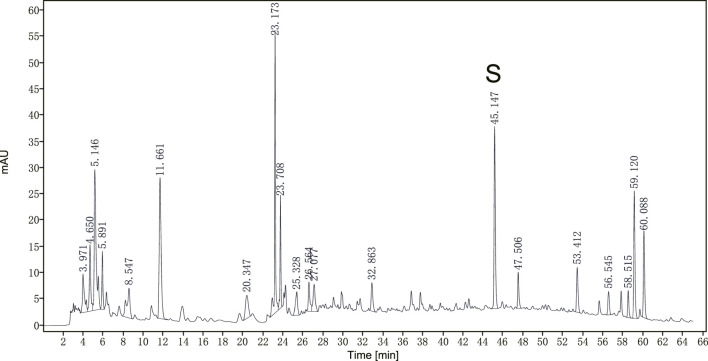
The optimized HPLC chromatogram of JPHTF monitored by a DAD detector at 260 nm. S: calycosin-7-O-b-D-glucoside.

A representative chromatogram of **JPHTF** was monitored by DAD detector, which revealed that the main compounds were eluted among the retention time of 4–12 min, 22–24 min, 44–46 min, 58–60 min, with calycosin-7-O-b-D-glucoside identified as one of the main compounds (Figure 1). The compounds of JPHTF are listed in Table 1.

### 3.2 Baseline characteristics of patients with stage II/III and IV colorectal cancer

A total of 300 patients with stage II/III CRC and 412 patients with stage IV CRC were included in the retrospective analysis. In the stage II/III cohort, patients were divided into placebo (*N* = 147) and TCM (*N* = 153) groups. In the stage IV cohort, patients received treatment with Western medicine (WM) (*N* = 120), TCM (*N* = 138), and integrated traditional Chinese and Western medicine (ITWM) (*N* = 154), respectively. The clinical characteristics of these patients are summarized in Table 2. In the stage II/III CRC cohort, the number of patients with rectal and colon cancer was similar, while in the stage IV CRC cohort, the number of patients with colon cancer exceeded the number of patients with rectal cancer. The majority of patients in the stage II/III CRC cohort were in a moderately differentiated state. Additionally, in the stage IV cohort, RAS or RAF status was detected in 232 patients.

**TABLE 2:** Clinicopathologic features of patients with CRC in stage II/III cohort and IV cohort.

Variable	II and III CRC cohort		Variable	IV CRC cohort		
	Placebo	TCM		WM	TCM	ITWM
Gender (*N* = 300)			Gender (*N* = 412)			
Male (*N* = 180)	89	91	Male (*N* = 260)	85	75	100
Female (*N* = 120)	58	62	Female (*N* = 152)	35	63	54
Age (Median)	61 (26–79)	59 (28–77)	Age (Median)	58 (31–78)	62 (18–81)	61 (35–81)
Histology (*N* = 300)			Histology (*N* = 412)			
Adenocarcinoma	133	140	Adenocarcinoma	112	125	143
Mucinous adenocarcinoma	9	6	Mucinous adenocarcinoma	3	6	4
Coexistent histologic type	5	5	Coexistent histologic type	4	4	5
Other	0	2	Other	1	3	2
Primary tumor location (*N* = 300)			Primary tumor location (*N* = 412)			
Rectum	73	78	Rectum	48	48	66
Colon	74	75	Colon	72	90	88
			Ascending	20	27	30
Nerve invasion and Vascular tumor emboli (*N* = 291)	11/142	17/149	Transverse	4	5	4
			Descending	3	13	13
			Sigmoid	38	37	38
			Rectosigmoid junction	6	7	2
			Descending and Sigmoid	1	1	1
Differentiation (*N* = 300)			RAS/RAF status (*N* = 232)			
Well differentiated	3	2	*KRAS* ^Mut^ (*N* = 226)	34/94	30/54	39/78
Moderately differentiated	102	109	*NRAS* ^Mut^ (*N* = 175)	8/78	0/43	6/54
Poorly differentiated	19	12	*BRAF* ^Mut^ (*N* = 177)	3/74	1/45	7/58
Undifferentiated	1	1				
Well and Moderately	1	1	No. of Metastases location (*N* = 412)			
Moderately and Poorly	10	13	≤1	68	48	55
Unknown	11	15	>1	52	90	99
			Metastases location			
			Liver	89	77	91
			Lung	43	82	77
			Liver and Lung	21	39	35

### 3.3 Therapeutic effects of JPHTF on patients with stage II/III CRC

In this study, we evaluated the improvement in quality of life for patients with stage II/III CRC using the TCM Symptom Classification Rating Scale, the Edmonton Symptom Assessment System (ESAS), and the Eastern Cooperative Oncology Group (ECOG) Performance Status Scale before and after treatment. The experimental group received JPHTF intervention, while the control group received a placebo that had a similar appearance to JPHTF. Both groups received three courses of treatment (1 month per course), followed by an initial follow-up after treatment and subsequent follow-ups every 3 months for a total of two visits, to evaluate the restorative effects of JPHTF on the functional recovery of stage II/III CRC patients after surgery and chemotherapy. Table 3 presents the results of the TCM symptom classification rating scale at baseline and during the three follow-up visits post-intervention. No significant difference was found in 10 syndromes between the placebo and TCM group at baseline (*p* > 0.05). However, abdominal distension in the TCM group showed significant improvement compared to the placebo group at the end of treatment and during subsequent follow-ups (At 3 months, *p* = 0.062; At 6 months, *p* = 0.022; At 9 months, *p* = 0.003). In the TCM group, patients with abdominal distension discomfort (score = 2) had been eliminated by the end of treatment. The number of patients with mild abdominal distension (score = 1) continued to decrease in subsequent follow-ups, while the proportion of patients without abdominal distension (score = 0) was significantly higher than the placebo group at 6 months (TCM vs. Placebo, 80.4% vs. 70.1%, *p* = 0.038) and 9 months (TCM vs. Placebo, 87.6% vs. 74.1%, *p* = 0.003). Additionally, TCM may have contributed to improving shortness of breath issues in stage II/III CRC patients with after surgery and chemotherapy. At the second visit, patients in the TCM group showed significant improvement in post-activity shortness of breath (score = 1) and eliminating shortness of breath (score = 0) compared to the placebo group (*p* = 0.024).

**TABLE 3:** Follow-up characteristics of TCM and placebo group based on TCM syndrome scale scores.

Syndrome	Score	Baseline		*P*	3 months		*P*	6 months		*P*	9 months		*P*
		Placebo (*n* = 147)	TCM (*n* = 153)		Placebo (*n* = 147)	TCM (*n* = 153)		Placebo (*n* = 147)	TCM (*n* = 153)		Placebo (*n* = 147)	TCM (*n* = 153)	
Abdominal distension	0	98	113	0.254	105	117	0.062	103	123	0.022*	109	134	0.003*
	1	42	37		37	36		40	30		34	19	
	2	7	3		5	0		4	0		4	0	
	3	0	0		0	0		0	0		0	0	
Fatigue	0	63	75	0.628	80	83	0.509	85	93	0.173	89	92	0.203
	1	63	62		55	63		47	53		46	56	
	2	19	15		11	7		15	7		11	5	
	3	2	1		1	0		0	0		1	0	
Pain	0	110	118	0.157	109	120	0.585	115	124	0.781	113	125	0.254
	1	33	35		36	32		31	28		34	27	
	2	4	0		2	1		1	1		0	1	
	3	0	0		0	0		0	0		0	0	
Dry stool	0	118	121	0.776	119	126	0.136	115	130	0.194	120	131	0.443
	1	21	26		18	24		23	20		21	20	
	2	7	6		9	3		8	3		5	2	
	3	1	0		1	0		1	0		1	0	
Insomnia	0	88	90	0.255	92	93	0.137	95	101	0.788	100	114	0.435
	1	36	45		38	47		35	38		33	26	
	2	23	16		17	10		17	14		14	13	
	3	0	2		0	3		0	0		0	0	
Loose stool	0	97	101	0.448	92	114	0.063	97	116	0.177	98	118	0.11
	1	29	38		37	31		35	30		34	29	
	2	14	10		10	6		9	5		9	4	
	3	7	4		8	2		6	2		6	2	
Loss of appetite	0	98	102	0.512	103	116	0.158	108	120	0.347	114	129	0.311
	1	36	43		34	34		31	30		27	19	
	2	12	8		9	3		7	3		5	5	
	3	1	0		1	0		1	0		1	0	
Knee and back soreness	0	84	101	0.409	91	106	0.543	99	111	0.7	105	117	0.826
	1	53	46		49	42		43	37		37	32	
	2	8	5		5	3		4	3		4	3	
	3	2	1		2	2		1	2		1	1	
Altered bowel habits	0	95	98	0.461	95	104	0.165	94	113	0.271	99	114	0.124
	1	26	36		29	36		36	27		32	28	
	2	24	18		21	10		16	12		15	7	
	3	2	1		2	3		1	1		1	4	
Shortness of breath	0	99	108	0.069	104	117	0.185	100	125	0.024*	105	126	0.087
	1	35	41		35	34		40	25		35	24	
	2	12	3		7	2		6	2		6	2	
	3	1	1		1	0		1	1		1	1	

*p-values were calculated using the Chi-square or Fisher’s exact method. Bold indicates statistically significant (*p* < 0.05).

**FIGURE 2:**
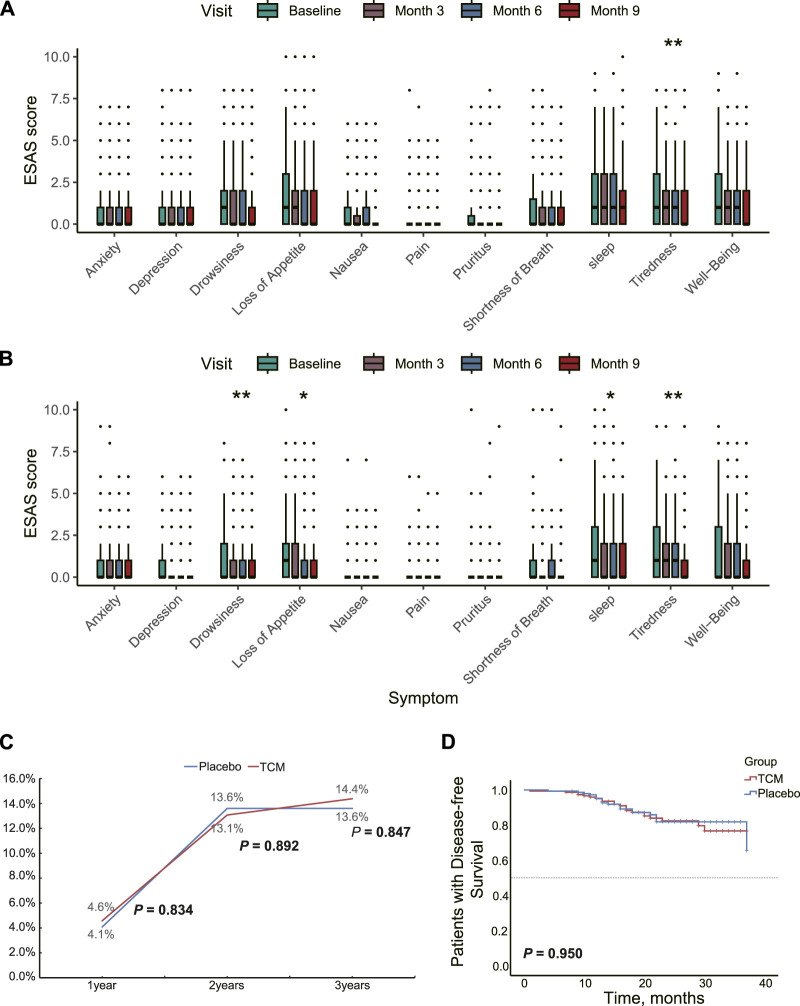
Therapeutic effects of JPHTF on patients with stage II/III CRC. Differences in symptom severity at baseline and during the three follow-up visits post-intervention based on ESAS score in placebo **(A)** group and TCM **(B)** group. **(C)** The recurrence and metastasis rate of patients in the placebo group and TCM group during the 3-year postoperative period. **(D)** Comparison of 3-Year Disease-Free Survival between Placebo and TCM Groups Using Kaplan-Meier Analysis. *p < 0.05; **p < 0.01; ***p < 0.001.

To multi-dimensionally evaluate the improvement in the quality of life for stage II/III CRC patients through JPHTF treatment, changes in the ESAS symptom severity were examined at baseline, the end of treatment, and during subsequent follow-ups. In the placebo group, a significant improvement in symptom severity was observed only for tiredness (*p* < 0.01, Figure 2A). However, compared to the placebo group, the TCM group showed significantly improved symptom severity for drowsiness, loss of appetite, sleep, and tiredness (*p* < 0.05, Figure 2B). The decreasing trend in ESAS scores for other domains or symptoms was more pronounced in the TCM group compared to the placebo group. Additionally, there was no significant difference in ECOG scores at baseline, the end of treatment, and subsequent follow-ups in both the TCM (*p* = 0.914) and placebo (*p* = 0.751) groups.

The preventive effect of JPHTF on post-operative recurrence was analyzed. 1-year, 2-year, and 3-year follow-ups after surgery showed that 13, 40, and 42 patients experienced recurrence or metastasis, respectively. There were no significant differences in recurrence rates between the TCM and placebo groups at these time points (*p* > 0.05, Figure 2C). Additionally, the median DFS at 3 years was not reached in either group, and no significant differences were found between the TCM and placebo groups (log-rank *p* = 0.750; Figure 2D).

### 3.4 Therapeutic effects of JPHTF on patients with stage IV CRC

**FIGURE 3:**
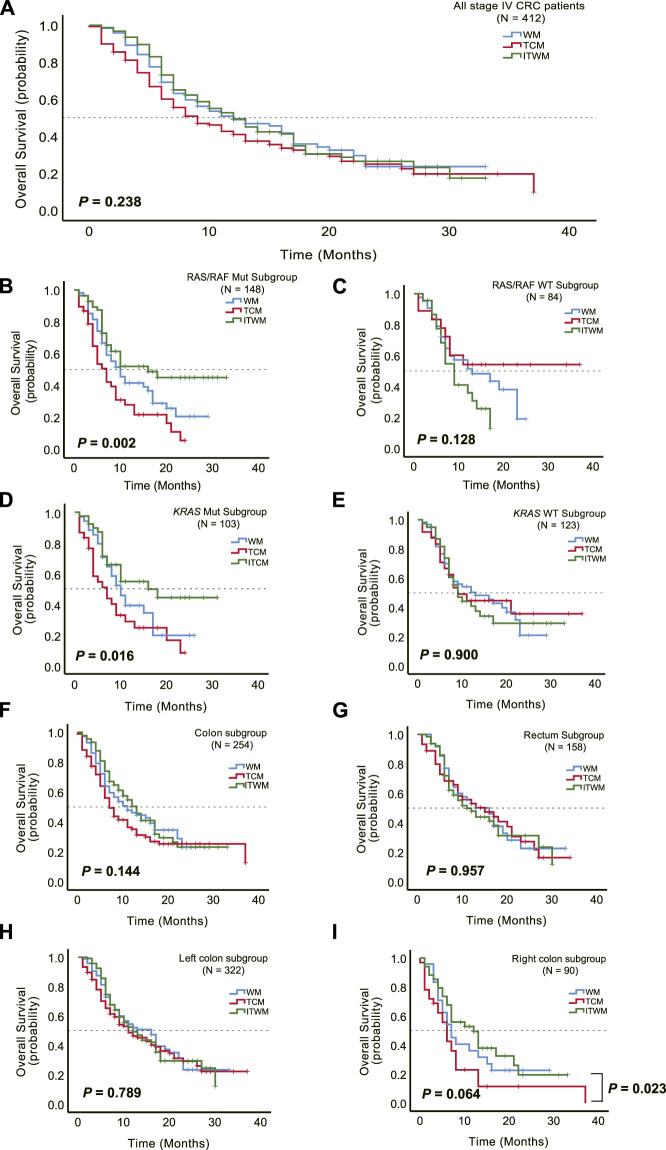
Therapeutic effects of JPHTF on patients with stage IV CRC. **(A)** Comparison of overall survival among Western medicine (WM) (*N* = 120), traditional Chinese medicine (TCM) (*N* = 138), and integrated traditional Chinese and Western medicine (ITWM) (*N* = 154) groups. Subgroup overall survival analysis was performed for RAS/RAF mutant (*N* = 148) **(B)** and wild-type (*N* = 84) **(C)**, *KRAS* mutant (*N* = 103) **(D)** and wild-type (*N* = 123) **(E)**, colon (*N* = 254) **(F)** and rectum (*N* = 158) **(G)**, left colon (*N* = 322) **(H)** and right colon (*N* = 90) **(I)**. *p < 0.05; **p < 0.01; ***p < 0.001.

To investigate the potential enhancement of survival outcomes in stage IV CRC patients, we conducted a retrospective analysis of the OS in a cohort of 412 individuals. These patients had varying degrees of metastasis. A total of 266 patients experienced endpoint events, with 75, 96, and 95 occurrences in the WM, TCM, and ITWM groups, respectively. The median follow-up duration for this analysis was 9.0 months (range, 1–37). Our findings revealed no significant difference in OS among the three treatment groups (median, WM vs. TCM vs. ITWM, 12.0 vs. 9.0 vs. 12.0 months; log-rank *p* = 0.238, Figure 3A).

Furthermore, we sought to ascertain the impact of JPHTF on the OS of CRC patients with specific genotypes. Among the selected 232 patients tested for RAS or RAF gene mutations, including 226 with *KRAS* mutations, a significant difference in OS emerged among the three groups. For those with RAS/RAF mutations, the patients treated by ITWM exhibited prolonged OS in comparison to the WM and TCM groups (median: WM vs. TCM vs. ITWM, 10.0 vs. 7.0 vs. 16.0 months; *p* = 0.002, Figure 3B). Similar observations were made in patients with *KRAS* mutations (median: WM vs. TCM vs. ITWM, 10.0 vs. 7.0 vs. 16.0 months; *p* = 0.016, Figure 3D).

However, no significant difference was discerned in RAS and RAF wild-type (WT) (median: WM vs. TCM vs. ITWM, 13.0 vs. not reached vs. 9.0 months; *p* = 0.128) or *KRAS* WT (median: WM vs. TCM vs. ITWM, 13.0 vs. 9.0 vs. 9.0 months; *p* = 0.900) patients (Figures 3C, E). Notably, in the RAS and RAF WT cohort, the TCM group exhibited a trend toward prolonged OS compared to the ITWM and WM groups, with a median OS not reached in the TCM group (Figure 3C).

Additionally, subgroup analysis by anatomic location (left versus right colon, rectum) revealed no significant difference in OS among the three groups (colon, median: WM vs. TCM vs. ITWM, 11.0 vs. 7.0 vs. 13.0 months; *p* = 0.144; rectum, median: WM vs. TCM vs. ITWM, 16.0 vs. 15.0 vs. 11.0 months; *p* = 0.957; left colon, median: WM vs. TCM vs. ITWM, 16.0 vs. 11.0 vs. 12.0 months; *p* = 0.789; right colon, median: WM vs. TCM vs. ITWM, 7.0 vs. 6.0 vs. 12.0 months; *p* = 0.064; Figures 3F–I). Nonetheless, in patients with right-sided colon cancer, treated by ITWM demonstrated a significantly longer OS compared to the TCM group (median: ITWM vs. TCM, 12.0 vs. 6.0 months; *p* = 0.023, Figure 3I), with a trend toward superiority relative to conventional Western medicine treatment as well.

### 3.5 Therapeutic efficacy of JPHTF in CRC PDX models

**FIGURE 4:**
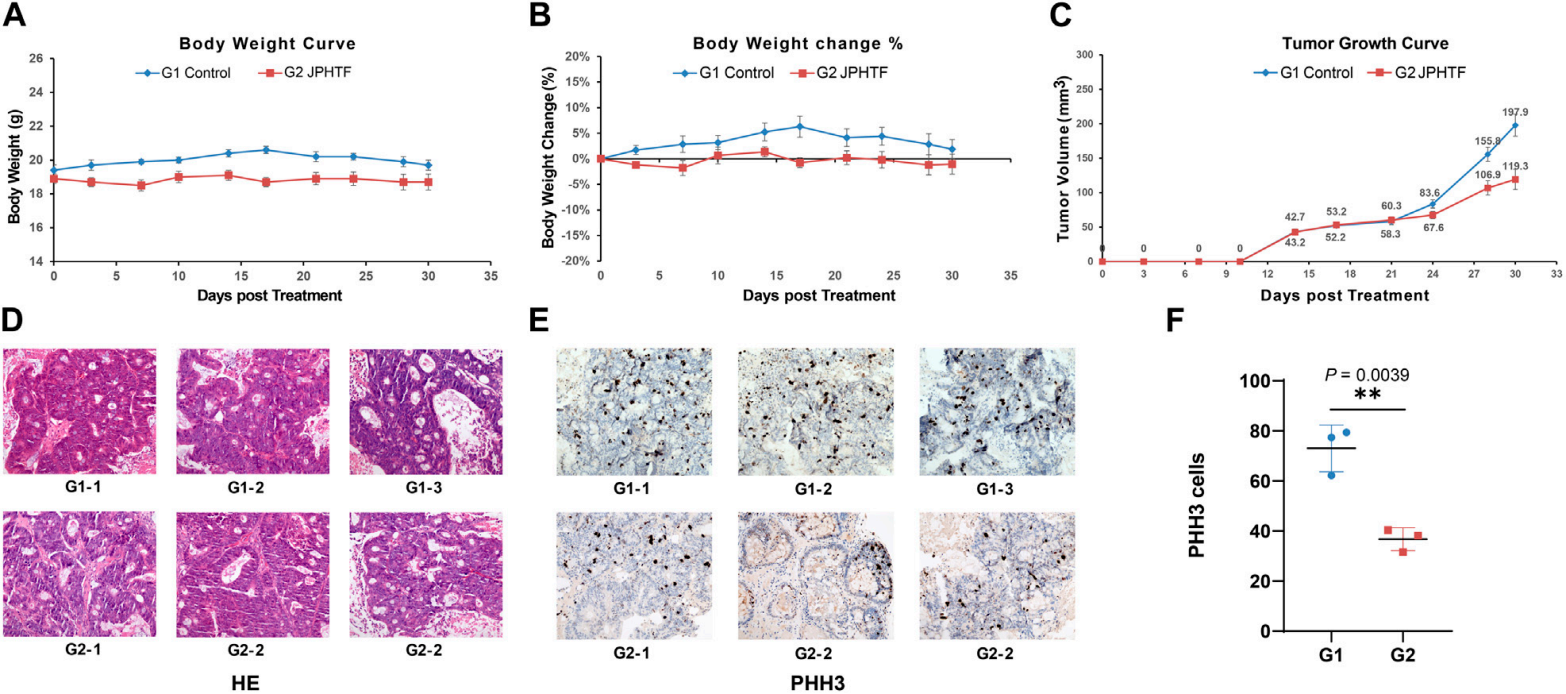
Therapeutic efficacy of JPHTF in CRC PDX mice model. Body weight **(A)** and Body weight change **(B)** of CRC PDX mice model. **(C)** The measured tumor growth. Hematoxylin and eosin staining **(D)** and the PHH3 expression **(E, F)** was performed in the untreated control group (G1) and JPHTF treatment group (G2). *p < 0.05; **p < 0.01; ***p < 0.001.

To verify the inhibitory effect of JPHTF on the growth of CRC tumors carrying RAS mutations, we injected a human colorectal cancer cell carrying *NRAS* mutations into two groups of mice. The untreated control group (G1) reached maximum average weight at day 17, followed by a continuous decline. In contrast, the JPHTF treatment group (G2) exhibited a relatively stable weight during the treatment period, showing no visible weight loss (Figures 4A, B). Starting from the tenth day after the injection of CRC cells, both groups showed an upward trend in tumor volume. At day 24, the tumor volume in the untreated control group (G1) surpassed that of the JPHTF treatment group (G2), with a nearly significant difference (*p* = 0.06). By day 28, the tumor volume in the untreated control group (G1) was significantly larger than that in the JPHTF treatment group (G2) (*p* < 0.01) (Figure 4C). Moreover, histopathological analysis of three selected mice from each group revealed a notably lower total tumor cells in the JPHTF treatment group (G2) compared to the control group (G1) (Figure 4D). Importantly, PHH3-positive tumor cells in the tumor tissue were significantly reduced in the JPHTF-treated experimental group (G2) compared to the untreated control group (G1) (*p* < 0.01) (Figures 4E, F).

### 3.6 Effect of JPHTF on the transcriptome of CRC PDX models

**FIGURE 5:**
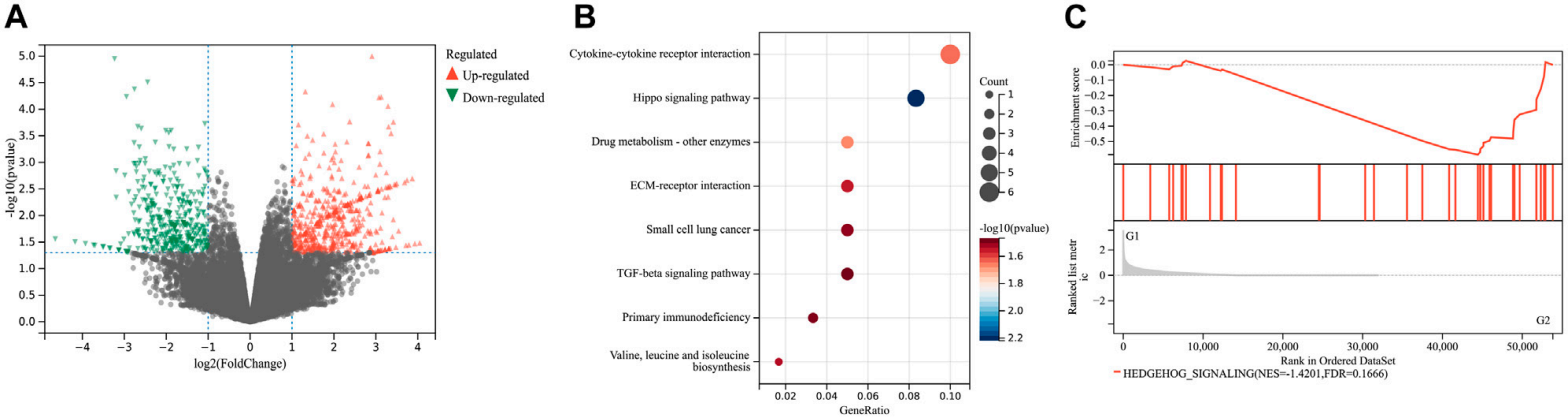
Effect of JPHTF on gene expression. **(A)** Volcano plot of differentially expressed genes between JPHTF treatment (G2) and control (G1) groups. Red dot, upregulated genes; Blue dot, downregulated genes; Gray dot, non-significantly expressed genes. **(B)** KEGG pathway cluster analysis based on the 600 upregulated DEGs as described in **(A)**. **(C)** The enriched pathways in JPHTF treatment group based on Gene Set Enrichment Analysis (GSEA).

To further investigate the impact of JPHTF on CRC biological functions, RNA-sequencing was performed on tumor tissues from mice, treated with or without JPHTF. A total of 53,837 differentially expressed genes (DEGs) (|log2 (FoldChange)| ≥ 1, *p*-value ≤0.05) were identified between JPHTF treatment (G2) and control (G1) groups, including 600 upregulated and 457 downregulated genes (Figure 5A).

Kyoto Encyclopedia of Genes and Genomes (KEGG) pathway enrichment analysis of the upregulated DEGs revealed significant enrichment in the Hippo signaling pathway (Figure 5B). Additionally, Gene Set Enrichment Analysis (GSEA) based on hallmark gene sets revealed a significant enrichment of the Hedgehog signaling pathway in the JPHTF-treated experimental group (G2), indicating regulation of this pathway by JPHTF (Figure 5C).

### 3.7 JPHTF may increase immune cell infiltration in CRC PDX models

**FIGURE 6:**
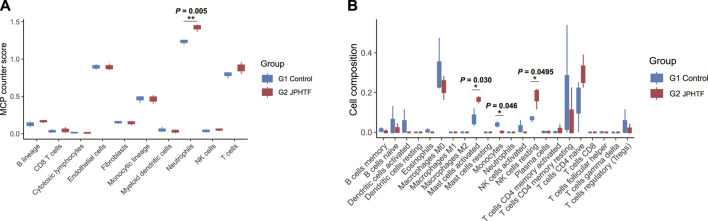
Identification of Immune cell infiltration associated with JPHTF groups. **(A)** MCP-Counter score differences of 10 immune-related cells between the G1 and G2 groups. **(B)** Immune cell infiltration differences of 22 immune-related cells between the G1 and G2 groups. *p < 0.05; **p < 0.01; ***p < 0.001.

To assess whether JPHTF treatment induces an immune response, we evaluated 10 and 22 immune-related cell types using the MCPcounter and CIBERSORT algorithms, respectively. MCPcounter analysis showed a significant increase in neutrophils in the JPHTF-treated experimental group (G2) compared to the untreated control group (G1) (*p* < 0.01, Figure 6A). CIBERSORT analysis revealed significant differences in 3 immune cell types between the two groups, with activated mast cells and resting NK cells significantly higher in the JPHTF-treatment group (G2), and monocytes significantly higher in the control group compared to the JPHTF-treated experimental group (G2) (*p* < 0.05, Figure 6B).

## 4 Discussion

The unique formula of JPHTF, a TCM, has been successfully utilized in clinical practice. To investigate the therapeutic efficacy of JPHTF, we conducted both retrospective analysis on different clinical cohorts and functional studies. The results suggested that the postoperative quality of life for stage II/III CRC patients was improved upon JPHTF administration, including reducing symptoms such as abdominal distension, shortness of breath, drowsiness, loss of appetite, sleep issues, and tiredness. Additionally, JPHTF, as an adjunct therapy in WM, prolonged OS in stage IV CRC patients with RAS/RAF mutations. Furthermore, JPHTF may have a better trend for OS in compared to the WM and ITWM groups in CRC RAS and RAF wild-type patients. Furthermore, JPHTF effectively suppressed tumor growth in the CRC PDX models carrying RAS mutations while concurrently maintaining the normal body weight of the mice. Remarkably, JPHTF treatment reduced tumor cell content in mice and decreased the PHH3 index. Transcriptome analysis indicated that JPHTF treatment potentially regulated Hippo and Hedgehog Signaling pathway and increased neutrophils, activated mast cells, and resting NK cells.

Traditional Chinese medicine intervention has unique advantages in various stages of colorectal cancer. Due to its minimal side effects and individualized treatment based on syndrome differentiation, traditional Chinese medicine is applied in adjuvant therapy for various tumors and in the combined use of traditional Chinese and Western medicine (Xiang et al., 2019). Although the recurrence rate after radical surgery in early-stage CRC patients is low, some patients still face the risk of recurrence and metastasis (Bastos et al., 2010; Xiong et al., 2023). High-risk stage II and III colorectal cancer patients often receive adjuvant chemotherapy to reduce the occurrence of recurrence and metastasis. However, these patients still have a high incidence of recurrence, metastasis, and cancer-related fatigue after treatment (Lu et al., 2019). Therefore, preventing recurrence and metastasis and improving the quality of life of patients after treatment have become the main tasks for the postoperative recovery of high-risk stage II and III colorectal cancer patients.

After a 3-month drug intervention for high-risk stage II and III CRC cohorts following radical surgery and 3–6 months of adjuvant chemotherapy, including the JPHTF group and the placebo group, no differences in DFS and recurrence rates were observed between the two groups. This may be due to the relatively short follow-up time and the inherently low recurrence rate in stage II/III CRC. However, the analysis of TCM syndrome scoring and ESAS scoring revealed that JPHTF significantly improved the quality of life of high-risk stage II and III colorectal cancer patients compared to the placebo group. This improvement included alleviation of symptoms such as abdominal distension, shortness of breath, drowsiness, loss of appetite, sleep, and tiredness, which are common postoperative symptoms affecting patients’ daily lives. From the perspective of traditional Chinese medicine, spleen-deficiency is the pivotal pathogenesis of colorectal cancer development (Sun et al., 2016). And symptoms commonly seen in patients with Spleen-deficiency, such as abdominal distension, tiredness, and loss of appetite, highlight the notable effectiveness of the JPHTF in treating II/III stage colorectal cancer with Spleen-deficiency syndrome. The improvement in these symptoms may be a result of enhanced patient immunity.

Interestingly, our immune infiltration analysis based on transcriptomics showed increased neutrophils, mast cells, and NK cells in the JPHTF treatment group PDX mice compared to the control group, indicating that JPHTF may activate the immune system. Previous studies have reported that traditional Chinese medicine can enhance tumor immunity, such as Phy906, a herbal extract based on Huangqin Tang, which enhanced the infiltration of M1 macrophages into tumors (Lam et al., 2015; Chang et al., 2020). Furthermore, Xihuang Wan was found to induce apoptosis of Treg cells in the tumor microenvironment of a 4T1 breast cancer mouse model through the upregulation of the MEKK1/SEK1/JNK1/AP-1 pathway (Su et al., 2018). Moreover, the ECOG score showed no significant differences between the TCM (*p* = 0.914) and placebo (*p* = 0.751) groups at baseline, treatment completion, and subsequent follow-up, indicating that the physical condition of high-risk stage II and III colorectal cancer patients is generally favorable.

Combining traditional Chinese medicine with Western medicine is a treatment option for advanced cancer patients. In particular, the combination of TCM with chemotherapy has become a primary approach for treating cancer. Chemotherapy could induce apoptosis in cancer cells, which inevitably leads to various adverse reactions such as gene mutations, cell toxicity, and drug resistance. TCM may play a crucial role in reducing the adverse effects of chemotherapy, thereby improving patient treatment outcomes and quality of life. Research has shown that Chinese herbal medicines such as curcumin, PHY906, and tonic Chinese medicine can reduce adverse reactions in advanced liver cancer patients treated with capecitabine and also improve the quality of life, anemia, and neutropenia in NSCLC patients after chemotherapy (Yen et al., 2009; Chen et al., 2010; Hsiao and Liu, 2010; Lam et al., 2010; Wang et al., 2011). Combining TCM with chemotherapy can effectively protect liver function, with Resveratrol improve cisplatin toxicity through an apoptosis-dependent mechanism (Liu et al., 2011; Liu et al., 2018). Additionally, ITWM could enhance the OS of breast cancer and CRC patients (Lee et al., 2020; Yeh et al., 2020). Our cohort demonstrated that patients carrying RAS/RAF mutations exhibited a superior OS in the ITWM group compared to others. This highlights the potential of JPHTF as a complementary approach to conventional Western medicine in extending survival for stage IV CRC patients and suggests its potential role in regulating the RAS/RAF signaling pathway. Importantly, JPHTF significantly inhibited tumor growth in PDX mice carrying RAS mutations. However, our transcriptomic analysis in the PDX models did not detect enrichment of related genes in this pathway, but instead found enrichment of the Hedgehog signaling and Hippo signaling pathways. The Hedgehog (Hh) signaling pathway has various roles in controlling cell proliferation, tissue patterning, stem cell maintenance, and development (Zhang and Beachy, 2023). It participates in the oncogenesis of CRC and regulates colonic enterocyte differentiation. High Hh-Smo-Gli activity is acquired in CRC for tumor cell survival and metastasis. Importantly, activation of the Hh signaling pathway, besides depending on canonical signaling (via Smo), can also be achieved through non-canonical activation (via the RAS/RAF pathway) in colon cancers (Varnat et al., 2009; Cai et al., 2015; Wu et al., 2017). The Hippo signaling pathway is a signaling cascade that controls organ size by regulating the activity of the nuclear transcription factors YAP/TAZ, controlling cell proliferation, apoptosis, and metabolism, and participating in tumor initiation and progression, metastasis, and drug resistance (Fu et al., 2022). Furthermore, the RAS/RAF signaling pathway can influence the normal regulation of cell proliferation and growth through the Hippo signaling pathway. Loss of the Hippo pathway may have a significant impact on tumors carrying RAS/RAF mutations (Kapoor et al., 2014; Lin et al., 2015). We hypothesize that the JPHTF may regulate the RAS/RAF pathway to inhibit Hedgehog signaling activation. It may also block the Hippo signaling pathway, enhancing the treatment effectiveness in CRC patients with RAS/RAF mutation, and suppressing tumor cell proliferation and metastasis. This association reveals the regulation of the body’s signaling pathways by JPHTF in CRC patients with RAS/RAF mutations, holding significant implications for understanding the mechanisms that inhibit tumor occurrence and development.

Although we were unable to validate whether TCM can reduce tumor volume in our two retrospective cohorts, the PDX model confirmed the efficacy of TCM in reducing tumor volume, suggesting that the JPHTF may inhibit tumor cell proliferation and induce apoptosis. The ginsenosides in the JPHTF can inhibit tumor growth by inducing apoptosis, suppressing proliferation, metastasis, and angiogenesis. Modern pharmacological research has discovered that ginsenoside Rh4 triggers apoptosis and autophagy in colorectal cancer cells by activating the ROS/JNK/p53 pathway, while ginsenoside Rb2 inhibits epithelial-mesenchymal transition in colorectal cancer cells through the TGF-β1/Smad signaling pathway. Additionally, purified ginsenoside compound K has been shown to regulate cell cycle transition and induce apoptosis, thus exerting therapeutic effects in colorectal cancer (Li et al., 2009; Wang et al., 2018; Wu et al., 2018; Dai et al., 2019).

Based on these findings, we aim to further investigate the impact of JPHTF on the body’s signaling pathways and immune response, and conduct long-term follow-up in larger cohorts to develop an adjunctive therapy that can effectively benefit CRC patients.

## Data Availability

The raw transcriptome data can be found in the GEO repository; accession number GSE269502 (available at: https://www.ncbi.nlm.nih.gov/geo/query/acc.cgi?acc=GSE269502).
