# Supplementary material for: Traditional Chinese Medicine JianPiHuaTan formula improving quality of life and survival in patients with colorectal cancer through RAS/RAF downstream signaling pathways
Source: Front Pharmacol. 2024 Jun 20;15:1391399. doi: 10.3389/fphar.2024.1391399 (PMC11225497; doi:10.3389/fphar.2024.1391399)
Supplement: Supplementary file 1 [file DataSheet1.pdf]

Supporting Information

Jian He 1†, Guojun Li 2†, Yu Wu 2, Tong Zhang 2, Mingjiang Yao 3, Mingxuan Zang 2, Jianhua Zou 2, Jinjie Song 2, Liusheng Li 4, Qian Chen 5*, Guang Cao 6* and Linlin Cai 2*

1 GCP Center, Guang'anmen Hospital, China Academy of Chinese Medical Sciences, Beijing, China,
2 Department of Oncology, Xiyuan Hospital, China Academy of Chinese Medical Sciences, Beijing, China,
3 Institute of Basic Medical Sciences of Xiyuan Hospital, China Academy of Chinese Medical Sciences, Beijing Key Laboratory of Pharmacology of Chinese Materia, Beijing, China,
4 Department of Oncology, Beijing Hospital of Integrated Traditional Chinese and Western Medicine, Beijing, China,
5 Thorgene Co., Ltd., Beijing, China,
6 Department of General Surgery, Beijing Anzhen Hospital, Capital Medical University, Beijing, China

† Jian He and Guojun Li, as co-first authorship, contributed equally to this work.

* Correspondence:

Qian Chen, chenqian@thorgene.com

Guang Cao, cguang2@163.com

Linlin Cai, 15911062978@163.com

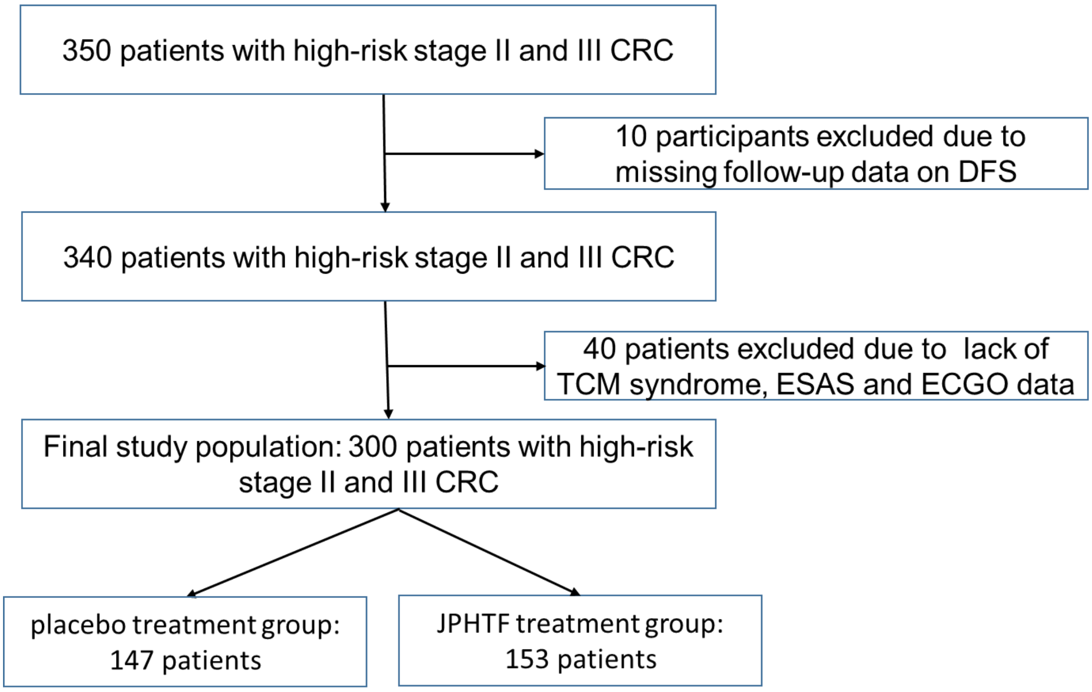

Figure S1. Flow-chart of high-risk stage II and III CRC patients' progress into the study steps.

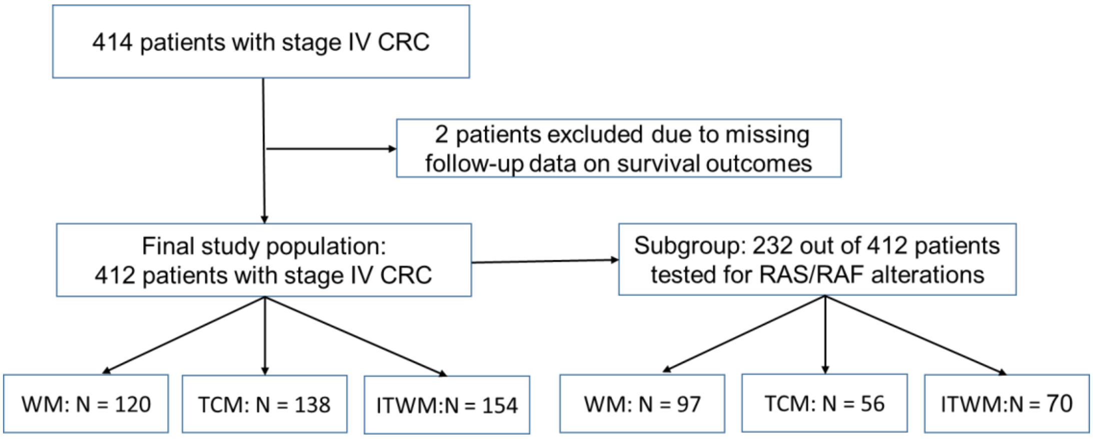

Figure S2. Flow-chart of stage IV CRC patients' progress into the study steps.

Table S1 JPHTF Granule Manufacturing Details

Botanical drug	TCM decoction pieces	Dosage equivalents (g/g)	Granule (g)
Mongolia Astragalus	30	2.5	12.0
Ginseng	10	2.5	4.0
Arisaema heterophyllum Blume	6	4.3	1.40
Ligustri Lucidi Fructus	10	2.6	3.85
Poria cocos	30	12.5	2.40
Epimedium	10	6.5	1.54
Turmeric	10	5.5	1.82
Salvia chinensis	15	6.7	2.24

Table S2 The Production Process of JPHTF.

Botanical drug	Ratio of botanical drugs to solvent	Extraction	Repetition times	Time(h)	Temperature (℃)	Solvents	Excipients	Functions of excipients
Mongolia Astragalus	1：10(1st)&8(2nd)	Decoct and boil	2	4	100	Water	Dextrin	Improve drug stability, solubility, adhesion
Ginseng	1：10(1st)&8(2nd)	Decoct and boil	2	2.5	100	Water	Dextrin	Improve drug stability, solubility, adhesion
Arisaema heterophyllum Blume	1：12(1st)&12(2nd)	Decoct and boil	2	4	100	Water	Dextrin	Improve drug stability, solubility, adhesion
Ligustri Lucidi Fructus	1：10(1st)&10(2nd)	Decoct and boil	2	4	100	Water	Dextrin	Improve drug stability, solubility, adhesion
Poria cocos	1：10(1st)&10(2nd)	Decoct and boil	2	4	100	Water	Dextrin	Improve drug stability, solubility, adhesion
Epimedium	1：12(1st)&12(2nd) &12(3rd)	Decoct and boil	3	4.5	100	Water	Dextrin	Improve drug stability, solubility, adhesion
Turmeric	1：10(1st)	Decoct and boil	1	6	95	Water	Dextrin/β-cyclodextrin	Improve drug stability, solubility, adhesion/ Reduce volatile oil volatilization
Salvia chinensis	1：12(1st)&12(2nd)	Decoct and boil	2	3	100	Water	Dextrin	Improve drug stability, solubility, adhesion

Selected chromatographic information during optimation was present in Figure S3-S6 along with their corresponding gradients (Table S3-S6).

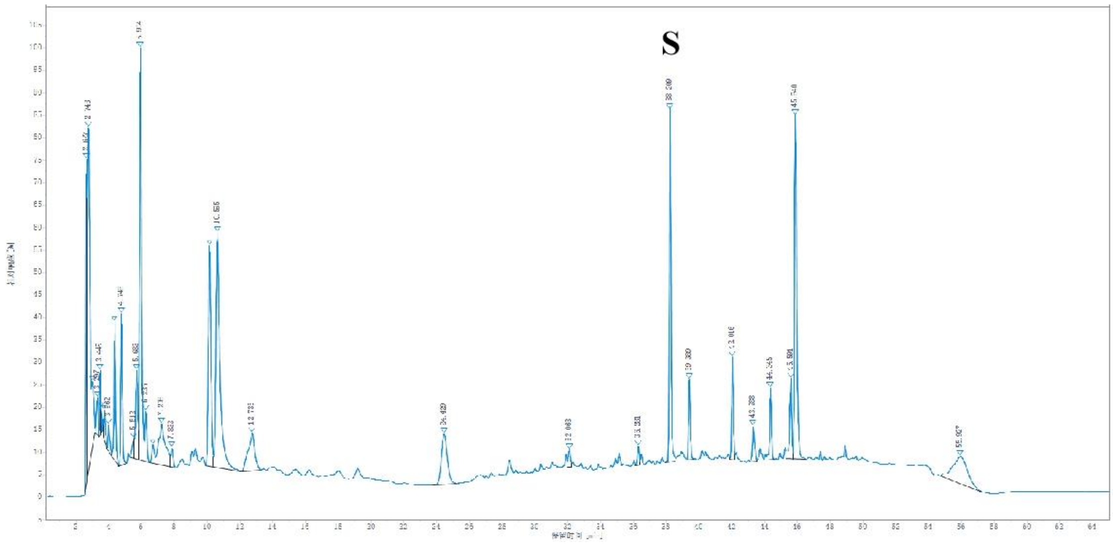

Figure S3 HPLC chromatogram of JPHTF eluted by gradient 1 (260 nm).

S:calycosin-7-O-b-D-glucoside.

Table S3 The elution procedure of gradient 1

Retention time（min）	A /water（%）	B /methanol（%）
0	90	10
40	40	60
45	5	95
50	5	95
55	90	10
65	90	10

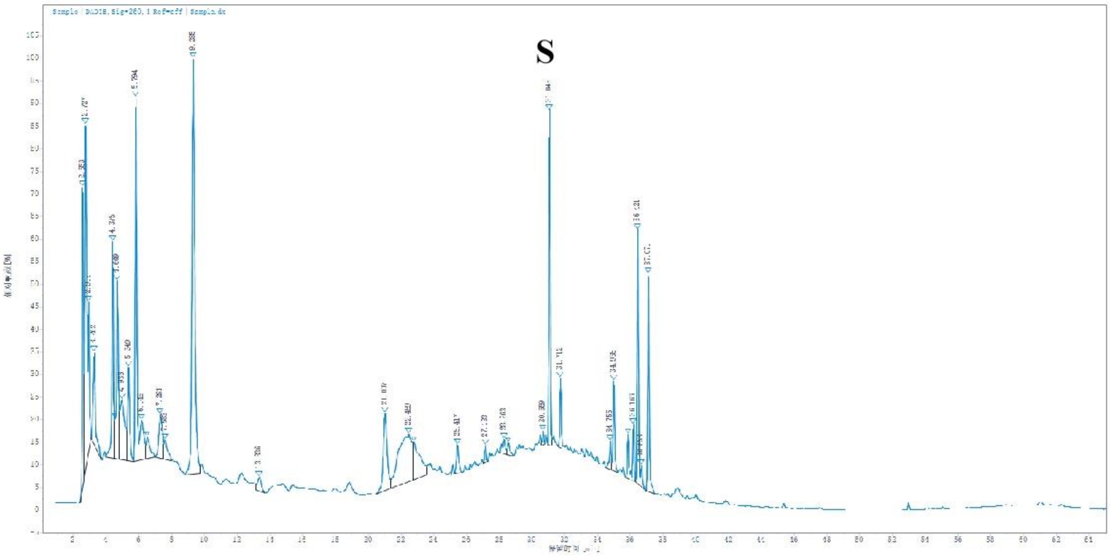

Figure S4 HPLC chromatogram of JPHTF eluted by gradient 2 (260 nm).

S:calycosin-7-O-b-D-glucoside.

Table S4 The elution procedure of gradient 2

Retention time（min）	A /water（%）	B /acetonitrile（%）
0	98	2
15	98	2
45	40	60
55	5	95
65	5	95
0	98	2

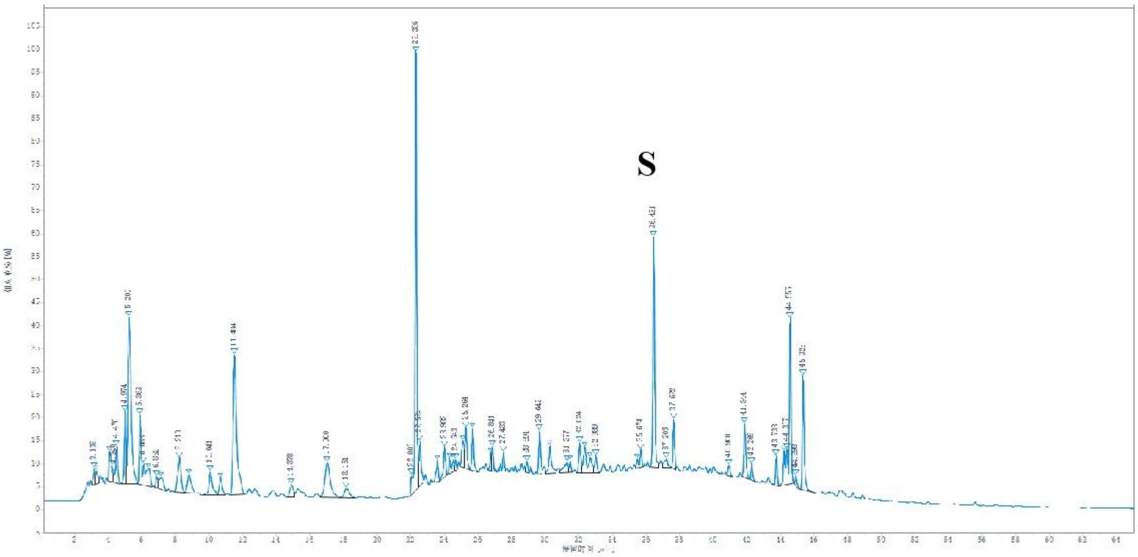

Figure S5 HPLC chromatogram of JPHTF eluted by gradient 3 (260 nm).

S:calycosin-7-O-b-D-glucoside.

Table S5 The elution procedure of gradient 3

Retention time（min）	A /water（%）	B /acetonitrile（%）
0	100	0
15	100	0
45	40	60
55	5	95
65	5	95

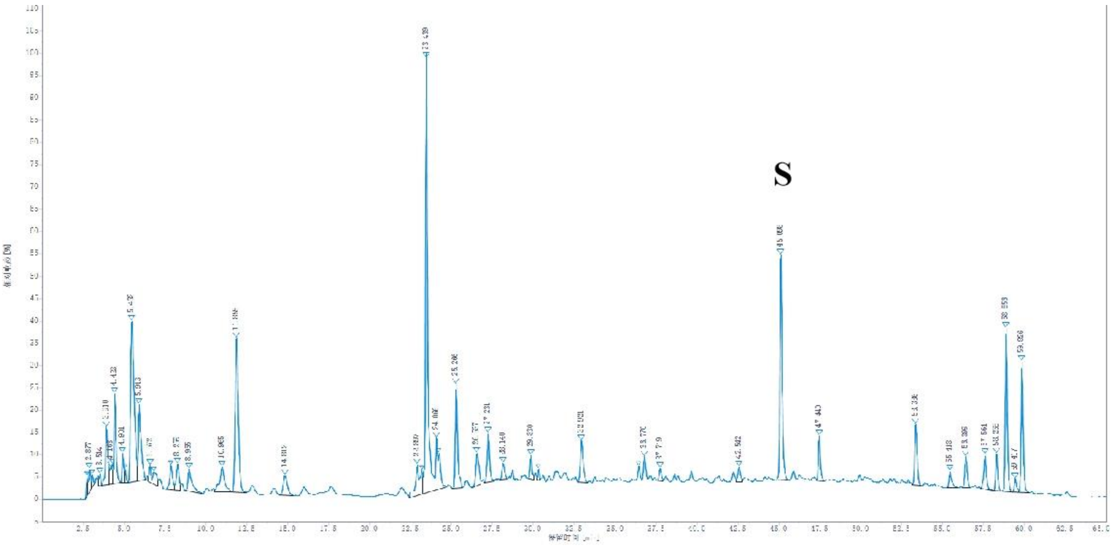

Figure S6 HPLC chromatogram of JPHTF eluted by gradient 4 (260 nm).
S:calycosin-7-O-b-D-glucoside.

Table S6 The elution procedure of gradient 4

Retention time（min）	A /water（%）	B /acetonitrile（%）
0	100	0
15	100	0
65	60	40
